# European survey on metabolic and cardiovascular risk in Cushing syndrome

**DOI:** 10.1007/s40618-025-02663-9

**Published:** 2025-09-06

**Authors:** Alessandro Mondin, Mattia Barbot, Filippo Ceccato, Alberto M. Pereira, Anna Angelousi, Atanaska Elenkova, Birute Zilaitiene, Camilla Schalin-Jäntti, Carlien De Herdt, Christina Kanaka-Gantenbein, Dominique Maiter, Federico Gatto, Gudmundur Johannsson, Kirstine Stochholm, Maria João Bugalho, Mario Detomas, Martin Reincke, Meropi Toumba, Roberta Giordano, Stylianos Tsagarakis, Susan M. Webb, Nienke R. Biermasz, Carla Scaroni

**Affiliations:** 1 Department of Medicine-DIMED, University of Padova, Padova, Italy; 2 Endocrinology Unit, University Hospital of Padova, Padova, Italy; 3 Center for Endocrine Tumors Leiden, Department of Medicine, Division of Endocrinology, Leiden University Medical Center, Leiden, The Netherlands; 4 Pituitary Centre Amsterdam, Amsterdam, The Netherlands; 5 Unit of Endocrinology, First Department of Internal Medicine, Laikon Hospital, Center of Excellence of Endocrine Tumours, National and Kapodistrian University of Athens, Athens, 11527 Greece; 6 Department of Endocrinology, Faculty of Medicine, Medical University-Sofia, USHATE Acad. Iv. Penchev, 2, Zdrave Str, Sofia, 1431 Bulgaria; 7 Institute of Endocrinology, Medical Academy, Lithuanian University of Health Sciences, Kaunas, 50161 Lithuania; 8 Endocrinology, Abdominal Center, Helsinki University Hospital and University of Helsinki, ENDO-ERN (European Reference Network on Rare Endocrine Conditions), Helsinki, Finland; 9 Department of Endocrinology, Diabetology and Metabolic Diseases, Antwerp University Hospital, Antwerp, Belgium; 10 Division of Endocrinology, Diabetes and Metabolism, Aghia Sophia ENDO- ERN Center for Rare Pediatric Endocrine Disorders, First Department of Pediatrics, Medical School, Aghia Sophia Children’s Hospital, National and Kapodistrian University of Athens, Athens, 11527 Greece; 11 Department of Endocrinology and Nutrition, Cliniques Universitaires Saint-Luc UCL, Bruxelles, Belgium; 12 Endocrinology Unit, Department of Internal Medicine and Medical Specialties, University of Genova, Genova, 16132 Italy; 13 IRCCS Ospedale Policlinico San Martino, Genova, 16132 Italy; 14 Department of Internal Medicine and Clinical Nutrition, Institute of Medicine, Sahlgrenska Academy, Department of Endocrinology, University of Gothenburg, Sahlgrenska University Hospital, Gothenburg, Sweden; 15 Department of Internal Medicine and Endocrinology, Aarhus University Hospital, Aarhus, Denmark; 16 Serviço de Endocrinologia, ULS Santa Maria, Lisboa, Portugal; 17 Department of Internal Medicine I, Division of Endocrinology and Diabetes, University Hospital, University of Würzburg, 97080 Würzburg, Germany; 18 Medizinische Klinik und Poliklinik IV, LMU University Hospital, LMU Munich, Munich, Germany; 19 School of Medicine, University of Nicosia, Nicosia, 2414 Cyprus; 20 American Medical Center, Strovolos, 2047 Cyprus; 21 Department of Biological and Clinical Sciences, University of Turin, Turin, Italy; 22 Department of Endocrinology, Diabetes and Metabolism, European Reference Network on Rare Endocrine Conditions (ENDO-ERN), Evangelismos Hospital, Athens, 10676 Greece; 23 Department of Medicine, Univ Autonoma Barcelona, Barcelona, Spain; 24 Research Center for Pituitary Diseases, Department of Endocrinology, Institut de Recerca Sant Pau (IIB-Sant Pau), Hospital S Pau, Barcelona, Spain; 25 CIBERER Unit 747, Instituto de Salud Carlos III, Madrid, Spain

**Keywords:** Cushing’s syndrome, ACTH, Cortisol, Cardiovascular risk, Arterial hypertension, Diabetes, Obesity, Vascular disease

## Abstract

**Background:**

Cushing’s syndrome (CS) is associated with increased metabolic and cardiovascular (CV) risk factors and morbidities. Evidence-based guidelines for the management of these issues in active or remitted CS are not available, so best practice is derived from guidelines developed for the general population. We aimed to evaluate the awareness and practice variation for CV comorbidities of CS across Reference Centres (RCs) of the European Reference Network on Rare Endocrine Conditions (Endo-ERN).

**Methods:**

A dedicated online survey was distributed from June 2022 to December 2022 to Endo-ERN RCs with recognized expertise in adrenal and/or pituitary diseases.

**Results:**

19 centres provided complete responses to the survey, accounting for an estimated pool of around one thousand chronically cared CS patients across Europe. Most ERN experts consider patients with CS at high CV risk irrespectively of remission status. Preoperative cortisol-lowering treatment was a common practice, especially for severe cases, and deemed effective in reducing CV risk by many. Most comorbidities were regularly evaluated at diagnosis and during follow-up, although a lack of provocative testing to diagnose diabetes (used only in 26% of RCs) was evidenced. A strict glycaemic control was encouraged although its target differed. On the contrary, a less stringent approach to dyslipidaemia and overweight emerged. Preferred initial compounds for patients presenting comorbidities were angiotensin converting enzyme inhibitors, metformin and statins; lifestyle changes were preferred over drugs to control weight excess after cure. Screening for asymptomatic vascular disease was performed routinely and regularly repeated during follow-up by only half of the centres. Important heterogeneity in some responses emerged, especially regarding the effect of remission or medical treatment on comorbidities and CV risk.

**Discussion:**

Our survey highlights the awareness of ERN experts on management of metabolic and CV risk factors or disease in CS. Most of them use the current European guidelines and apply strategies for high CV risk patients, although not all these recommendations were fully followed. Since several CV risk factors seem to persist after disease remission, they should be adequately and promptly addressed. Population-specific studies are required to identify the optimal management of CV and metabolic comorbidities of CS patients.

**Supplementary Information:**

The online version contains supplementary material available at 10.1007/s40618-025-02663-9.

## Background

Endogenous Cushing’s syndrome (CS) is a rare condition with excessive cortisol secretion, either from an adrenal source or due to ACTH overproduction by a pituitary or ectopic source [1]. Overt hypercortisolism usually leads to the classical CS phenotype, whilst a mild disease may present with a more blurred clinical picture that complicates the diagnostic process [2]. Cortisol excess is also usually associated with a plethora of comorbidities such as metabolic syndrome, osteoporosis, infections, venous thromboembolism and various complications that decrease the quality of life and increase the overall mortality rate of these patients [3]. Prompt diagnosis and treatment are of the utmost importance to effectively reduce mortality and improve the quality of life.

As recently pinpointed by a metanalysis on the topic, cardiovascular (CV) events are the leading cause of mortality in CS patients [4]. Nevertheless, condition-specific recommendations for the management of CV risk factors in CS are lacking. Current guidance is provided by guidelines used in the general population neglecting important aspects of steroid excess. The most recent Consensus on Cushing’s Disease (CD) advises to evaluate, monitor and treat CV comorbidities of patients with hypercortisolism according to general population’s current guidelines and to consider CS patients as having high CV risk [5]. The importance of a long-term management plan of CV risk factors is further stressed by the fact that remission from cortisol excess may not fully revert CV risk [6, 7]. Better condition-specific management would also be of relevance to the more prevalent situation of exogenous hypercortisolism (steroid use).

Thus, our aim was to assess the standard of care for metabolic and cardiovascular risk factors and complications in CS across Europe through a survey directed to Reference Centres (RCs) of the European Reference Network on Rare Endocrine Conditions (Endo-ERN) among endocrinology units with recognized expertise in adrenal and/or pituitary diseases. The survey was designed to assess expert practitioners’ perception of CV risk in CS as well as to explore the modalities of screening and treatment of arterial hypertension, glucose homeostasis impairment, dyslipidaemia, overweight/obesity and vascular disease.

## Methods

An online survey with both single/multiple choice questions and open field queries was elaborated *(Supplementary Table 1)*. The resulting questionnaire was sent to all RCs that obtained endorsement by Endo-ERN in pituitary and/or adrenal Main Thematic Groups. A total of 71 centres were reached, and the questionnaire was circulated in two appeals, with a final deadline for compilation on 15th December 2022.

The survey covered various domains (epidemiology and definitions, CV risk assessment, arterial hypertension, diabetes, dyslipidaemia, vascular disease, obesity, and miscellaneous) for a total of 49 questions, some presenting sub-questions. Thromboembolic events in CS were previously addressed by another survey [8]. Outcomes of the investigation are summarized below.

We also conducted a post-hoc analysis assessing the degree of consensus reached in the single-choice closed questions. We evaluated the distribution of the answers and expressed them as percentages. Based on the percentage of the most chosen answer, we assigned the following evaluations on the agreement degree: >80%: consensus reached; 50–80%: majority reached; <50%: general disagreement or area of uncertainty.

Proportion of responses are expressed as counts and/or percentages. In case of quantitative data, medians and interquartile ranges have been calculated. We also performed simple linear regression analyses with SPSS statistics (IBM Corp. Released 2016. IBM SPSS Statistics for Windows, Version 24.0. Armonk, NY: IBM Corp.), with a significance threshold set at *p* < 0.05.

## Results

**Fig. 1:**
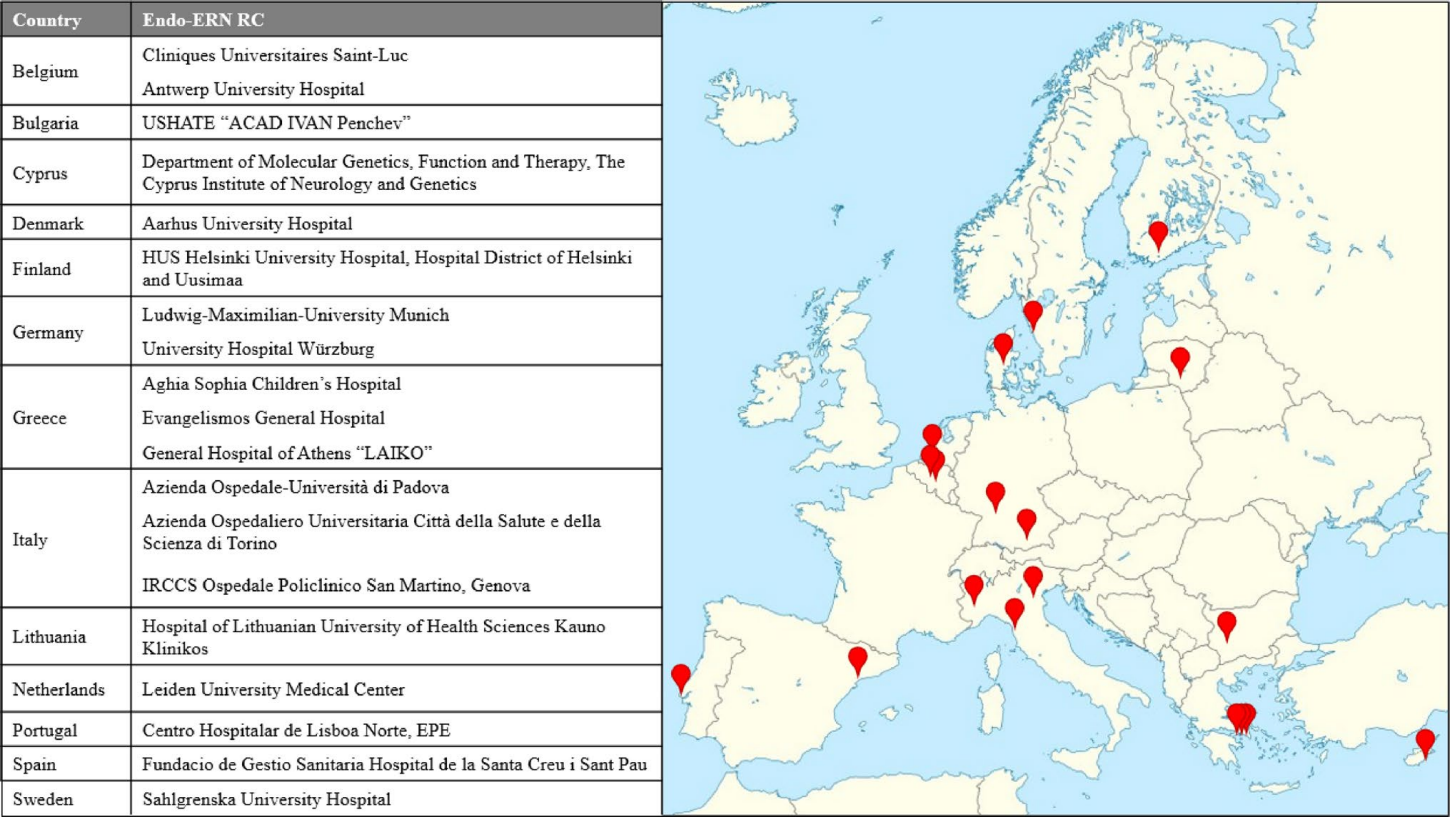
Geographical distribution of responding reference centres (RCs) across Europe. Endo-ERN: European Reference Network on Rare Endocrine Conditions; RC: Reference Centre

Following the second call for survey compilation, a total of 19 centres in 13 countries provided complete responses to the survey queries and were included in the analysis of results. Responding RCs were located homogenously across Europe, despite the lack of coverage of some countries. Four countries had multiple responding centres (*n* = 3 from Italy, *n* = 3 from Greece, *n* = 2 from Belgium and *n* = 2 from Germany) (Fig. 1).

### Local management

RCs provided an estimation of the pool of patients followed in 2020 and 2021, accounting for around one thousand chronically cared patients and around two hundred new diagnoses per year (Table 1) [9]. Most of the data are managed by internal databases (14/19), and 9 RCs also take part to the international registry ERCUSYN [10] (*Supplementary Fig. 1*). The two German centres also participate to a national database including neuroendocrine tumours (NeoExNET). Regarding follow-up, most of participants (14/19, 74%) agreed that a life-long monitoring is necessary, independently of the aetiology of CS. Vice versa, some RCs responded that in specific settings the discharge of a patient is possible, especially those with unilateral benign adrenal CS after curative surgery. The usual biochemical work-up for CS patients (independently of the aetiology) at diagnosis and during follow-up is also reported in *Supplementary Fig. 2*.

**Table 1:** Number of CS patients newly diagnosed and under chronic care. Both total and median number per center and interquartile ranges (within the squares) are reported. These numbers may depend on the definitions used by centres to define new and chronically cared patients

		Newly diagnosed CS patients		CS patients under chronic care	
Condition	Year	Total	Per center	Total	Per center
Cushing’s disease	2020	94	4 [2; 7]	631	8 [5; 49]
	2021	104	5 [2; 7]	656	5 [5; 48]
Ectopic ACTH secretion	2020	21	1 [0; 2]	59	2 [0; 3]
	2021	22	1 [0; 2]	63	1 [0; 4]
Benign adrenal CS	2020	72	2 [1; 5]	167	3 [1; 20]
	2021	84	2 [1; 5]	189	4 [1; 20]
Malignant adrenal CS	2020	32	1 [0; 3]	84	1 [0; 5]
	2021	35	1 [0; 4]	87	1 [0; 5]

CS: Cushing’s syndrome; ACTH: adrenocorticotropic hormone

### Cardiovascular risk

Participants unanimously agreed that CV risk is increased in CS, but whether this risk could be reversed by effective treatment was considered controversial. Indeed, around two third of responders (13/19, 68%) stated that patients with CS will always present increased CV risk. Six centres, on the contrary, identified the following high-risk phases during the course of the disease: (a) active phase prior to diagnosis (*n* = 6), (b) patients waiting for surgery (*n* = 5), (c) patients with active CS receiving medical treatment (*n* = 3), (d) in the years following effective surgery (up to 1 year for three centres, up to 5 years for 2 participants). According to the majority of participants, CV risk is related to the degree of cortisol excess (15/19, 79%). To stratify the severity of disease, all participants agreed on the pivotal role of urinary free cortisol (UFC) but most of them also suggested the use of late-night salivary cortisol (LNSC); other suggested parameters are reported in *Supplementary Fig. 3*.

Preoperative treatment with cortisol-lowering medications was thought to decrease CV complications in the peri-operatory period by 15/19 responders (79%). It was mostly applied only in case of severe hypercortisolism (11/19, 58%), although some centres indicated its routine use in CS patients (7/19, 37%); one centre reported the non-use of preoperative treatment. In this setting steroidogenesis inhibitors were the treatment of choice, especially ketoconazole (10/18, 56%), followed by metyrapone (6/18, 33%) and osilodrostat (2/18, 11%), a choice also depending on drug’s availability in the different countries before 2023. Smoking habit was usually assessed in CS patients (18/19, 95%). Concomitant CV risk factors (e.g. arterial hypertension, dyslipidaemia, diabetes, obesity, smoking habit) or a positive familial history could influence the work up for the patient (15/19, 79%): the endocrinologists were more prone to ask for more instrumental exams (9/15, 60%), to refer the patient to the cardiologist (5/15, 33%) or to consider a preoperative treatment to reduce cortisol levels (1/15, 7%).

### Arterial hypertension

**Fig. 2:**
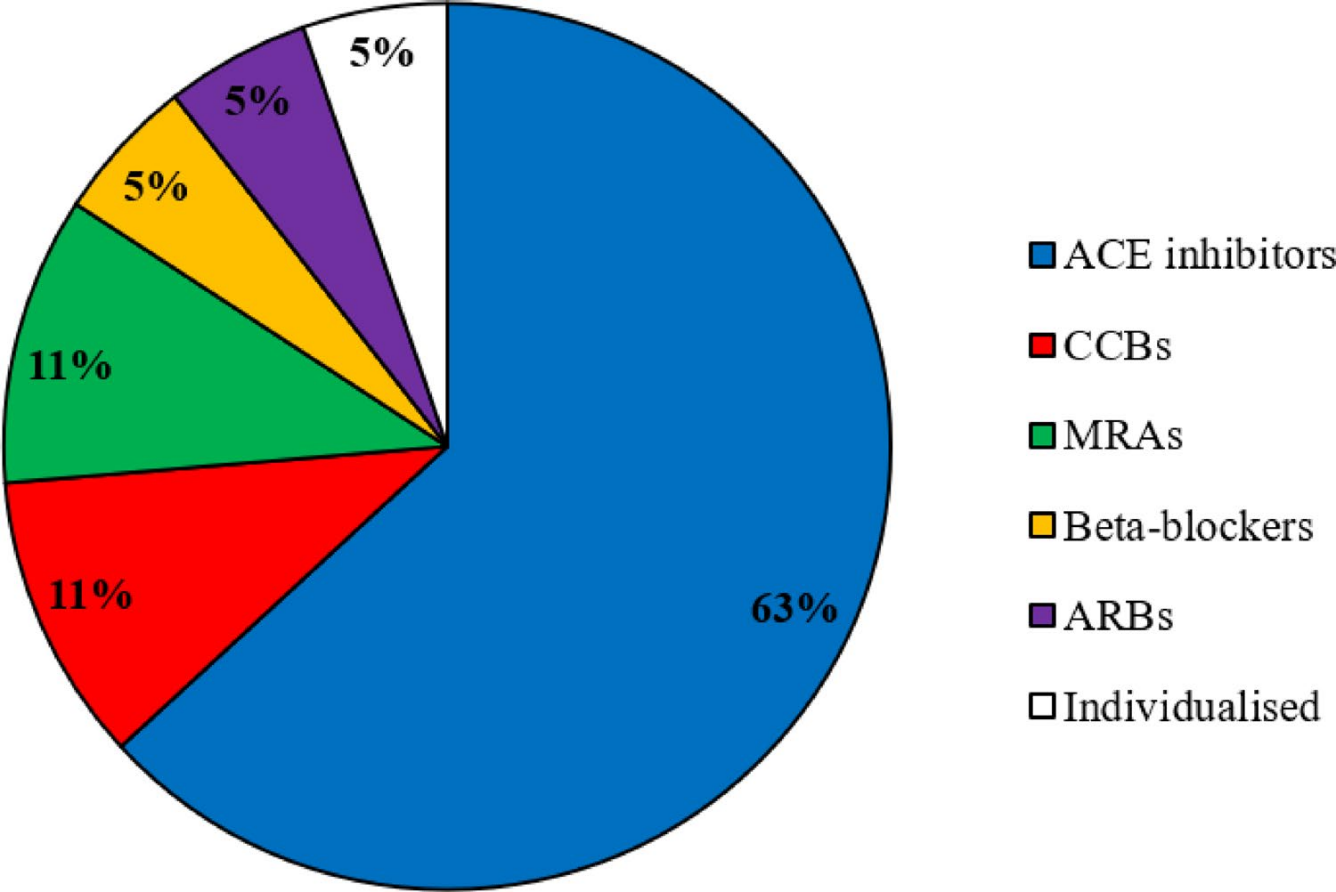
Survey results regarding first-line anti-hypertensive treatment. ACE: angiotensin converting enzyme; CCB: calcium-channels blocker; MRA: mineralocorticoid receptor antagonist, ARBs: angiotensin receptor blockers

All centres assessed the presence of arterial hypertension. However, the preferred tools for the evaluation varied among responders. Most centres agreed that blood pressure should be evaluated regularly at diagnosis and during follow-up in both remittent and persistent CS (17/19, 90%), two assessed it only at diagnosis. Diagnostic work up for arterial hypertension in RCs is reported in Table 2. Angiotensin converting enzyme inhibitors are by far the most used first-line treatment according to our responders (Fig. 2), with target blood pressure mainly set below 130/80 mmHg (Table 2).

**Table 2:** Responses distribution regarding comorbidities diagnosis and treatment targets

Domain	Topic	Options	Number of RCs (% if single choice query)
Arterial hypertension	Routine diagnostic parameters	*At clinic measures* Single measure Mean of three measures Both sitting and standing	19 9 3 3
		At home measures	11
		24 h monitoring	2
		Echocardiography	11
	SBP target	<120 mmHg	5/19, 26%
		<130 mmHg	11/19, 58%%
		<140 mmHg	2/19, 11%
		Individualized	1/19, 5%
	DBP target	<80 mmHg	16/19, 84%
		<90 mmHg	2/19, 11%
		Individualized	1/19, 5%
Glucose homeostasis	Routine diagnostic parameters and other related assessments	Fasting serum glucose	18
		HbA1c	18
		OGTT	5
		HOMA index	5
		2 h post-prandial glucose	3
	HbA1c target	<53 mmol/mol	6/18, 33.3%
		<48 mmol/mol	8/18, 44.4%
		<42 mmol/mol	4/18, 22.2%
Overweight/obesity	Preferred diagnostic assessment	BMI	14/19, 74%
		Waist to hip ratio	1/19, 5%
		Body composition Bioimpedance analysis X-ray analysis	3/19, 16% 2 1
		All of the above	1/19, 5%
Dyslipidaemia	Treatment threshold	LDLc >3.9 mmol/L	13/19, 68%
		LDLc >2.6 mmol/L	1/19, 5%
		Total cholesterol >5.2 mmol/L	1/19, 5%
		Triglycerides >1.7 mmol/L	1/19, 5%
		Individualized	2/19, 11%
	LDLc target on treatment	<2.6 mmol/L	11/19, 58%
		<1.8 mmol/L	6/19, 31%
		Individualized	2/19, 11%

RC: reference center; SBP: systolic blood pressure; DBP: diastolic blood pressure; HbA1c: glycosylated haemoglobin; OGTT: oral glucose tolerance test; BMI: body mass index; LDLc: calculated low-density lipoproteins

### Glucose homoeostasis impairments

**Fig. 3:**
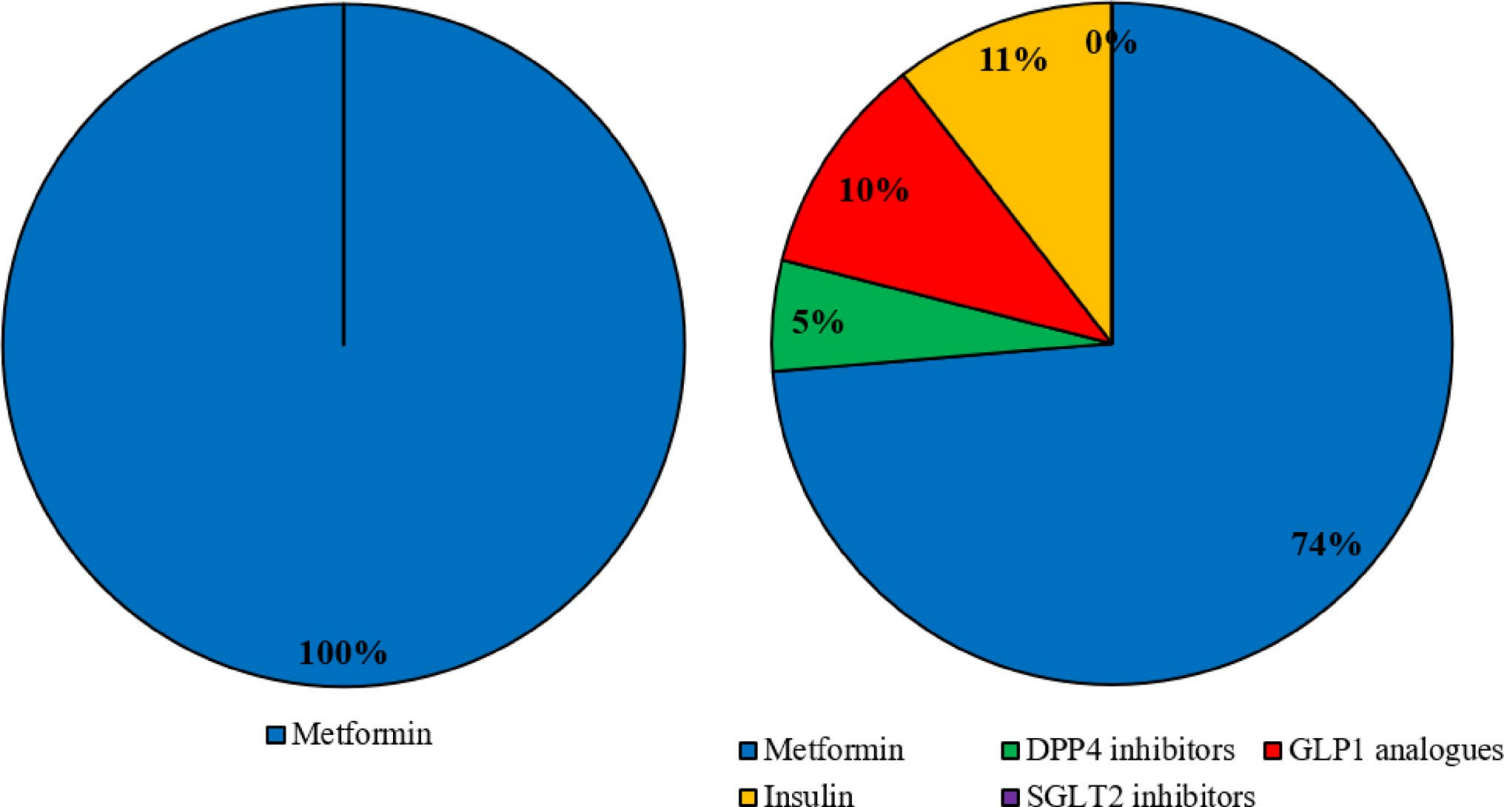
Survey results regarding first-choice anti-diabetic treatment at diagnosis (left) and for recurrent/persistent CS (right). DPP: dipeptidyl peptidase; GLP: glucagon-like peptide; SGLT sodium-glucose transporter

All participants reported glucose metabolism evaluation on a regular basis both at diagnosis and regularly during follow-up for persistent and remittent CS. The main parameters to assess it at diagnosis were fasting serum glucose and glycosylated haemoglobin (HbA1c) as reported in Table 2. Familial history of diabetes mellitus (DM) was inquired by most (79%). Metformin was unanimously pointed as first-line treatment for DM in *de novo* CS. In case of non-surgically treated patients or persistent/relapsed CS other compounds were listed, although metformin remained the prevailing option (Fig. 3). Among responders, 7 out of 19 (37%) reported local limitations on which anti-diabetic agents could be prescribed. Nevertheless, even in case of unrestricted choice the majority of them confirmed metformin as the preferred drug (57%, 4/7), while 3 centres addressed other preferable alternative initial compounds (glucagon like peptide (GLP) 1 analogues *n* = 2, sodium glucose transporter (SGLT) 2 inhibitors *n* = 1). Designed treatment targets are reported in Table 2.

### Dyslipidaemia

All but one centre agreed on a mandatory screening for dyslipidaemia at CS diagnosis and to repeat it regularly during follow-up in both persistent and remitted patients (95%). Thresholds to start treatment as well as calculated low-density lipoprotein (LDLc) targets are reported in Table 2. A medical treatment with statins was deemed appropriate ab initio by most centres (15/19, 79%), while others suggested a first attempt based on lifestyle changes. Most RCs (17/19, 90%) considered statin withdrawal after surgical remission and reassessment of the cardiovascular risk. Four of them (21%) performed this reassessment routinely 3 months after surgery, while others performed it only in case of long-lasting remission (i.e., at least one year, *n* = 6) or in case of concomitant comorbidities reversal after cure (7/19, 37%).

### Obesity

Obesity in CS patients was mainly assessed using body mass index (BMI), as showed in Table 2. For patients in remission with persisting overweight or obesity, lifestyle changes and physical exercise (18/19, 95%) were preferred over anorectic drugs.

### Sleep Apnoea

Sleep apnoea was evaluated by a minority of RCs (4/19, 21%), mostly at baseline only (3/4, 75%) and preferring the use of polysomnography (100%).

### Clinically silent cardiovascular disease

All participants agreed that CS carries high risk for CV disease. Nevertheless, CV diseases may become clinically evident (e.g., acute coronary syndrome, stroke) after going unnoticed for many years. Just around half of the centres performed routine screening for an early detection of silent CV disease in all patients (10/19, 53%); in some RCs this screening was justified in case of active CS (2/19, 11%) or in the presence of known risk factors (3/19, 16%). Four centres did not perform any screening for silent CV disease. Among the 15 RCs performing this screening, electrocardiogram (*n* = 13) followed by echocardiography (*n* = 11) and/or carotid ultrasound (*n* = 9) were the most used tools. All centres performed these assessments at diagnosis (*n* = 15); 9 of them also reassessed them throughout the follow-up independently of disease status, while 3 centres considered a re-testing only in case of active CS (*Supplementary Fig. 4*).

Antiplatelet treatment was mainly considered in case of documented vascular disease (58%). Some responders performed it in all CS (2/19, 11%) or for diabetic CS (3/19, 16%). Three centres found this treatment inappropriate due to bleeding concerns.

### The effect of hypercortisolism treatments on comorbidities

**Fig. 4:**
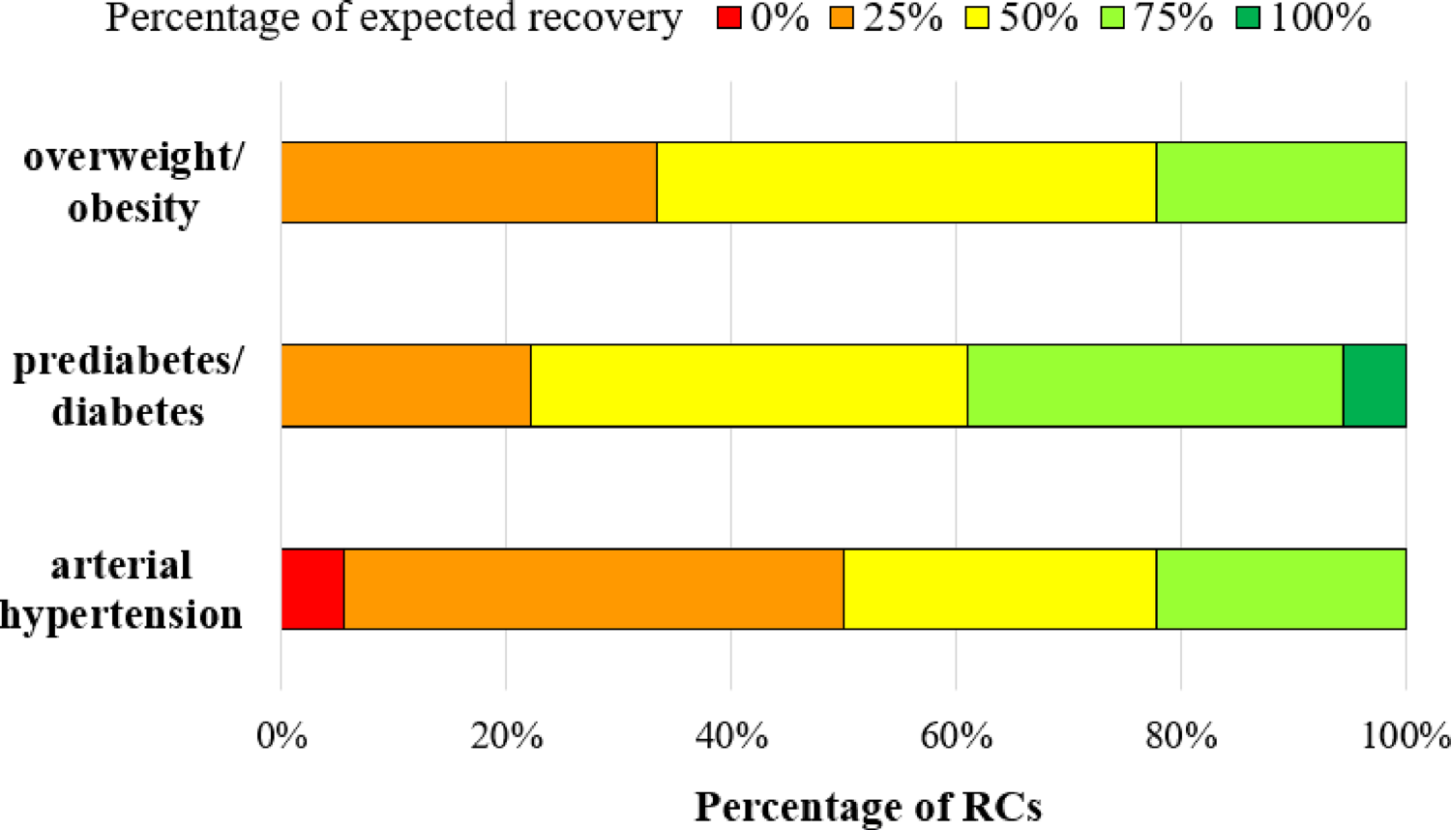
Percentages (%) of expected complete recovery (i.e., patients without criteria for the comorbidity and no ongoing treatment for it) from comorbidities after effective surgery

According to most of our responders (14/18, 78%), arterial hypertension reverted in only half of the cases (Fig. 4) after an effective surgery; nevertheless, there was general agreement on a significant reduction in the number/dosage of hypertensive drugs, with 33% of centres reporting a reduction in over 75% of cases and 39% in 50–75% of cases. Regarding medical treatment, anti-hypertensive drugs reduction was expected in about half of patients (50% expected a reduction in 25–50% of cases, 44% in 50–75% of patients).

As far as prediabetes/diabetes were considered, their expected remission rate after surgery varied across RCs. Most participants presumed a glucose homeostasis recovery in 50% or more of the successfully treated cases (Fig. 4).

According to 78% (14/18) of participants, at least half of the patients remain overweight/obese after surgical remission (Fig. 4); the percentage raised to 89% in case of persisting disease despite ongoing medical treatment (16/18).

### The effect of comorbidities on hypercortisolism treatment choice

The presence of arterial hypertension during active CS affected medical treatment’s choice for some physicians (10/19, 53%), generally guiding towards steroidogenesis inhibitors, sometimes as a part of combined approaches (*Supplementary Fig. 5*).

Twelve centres (63%) reported that the presence of DM influences cortisol-lowering medications choice towards steroidogenesis inhibitors (*Supplementary Fig. 5*).

### Degree of consensus analysis

**Fig. 5:**
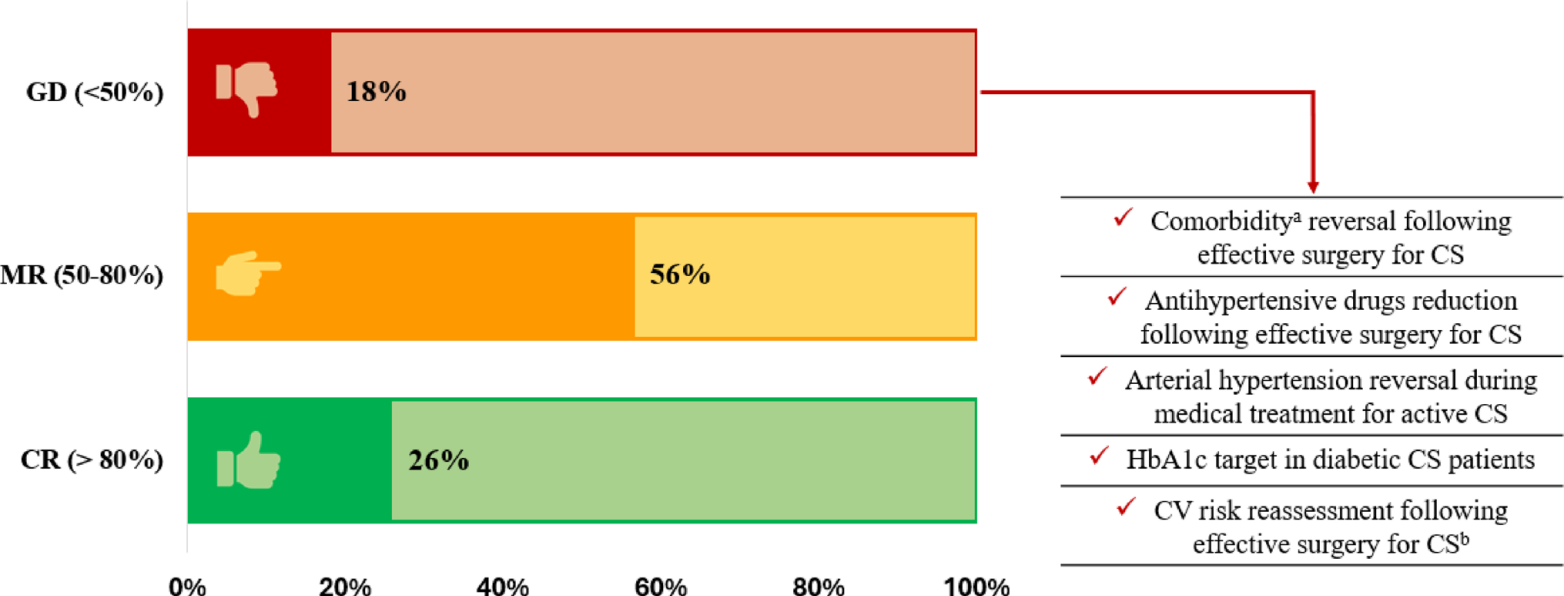
Evaluation of consensus degree (see methods). ^a^ Arterial hypertension, impaired glucose homeostasis and overweight/obesity. ^b^ The majority of responders performed the reassessment, but the premises and timing were heterogeneous. CR: consensus reached; MR: majority reached; GD: general disagreement; CS: Cushing’s syndrome; HbA1c: glycosylated haemoglobin; CV: cardiovascular

Among questions eligible for analysis (*n* = 39, including sub-questions), 7 presented a general disagreement. In the other cases there was a dominant response, but only in 10 cases a consensus (i.e., > 80%) was reached (Fig. 5).

Disagreement mainly regarded the effects of CS remission on comorbidities. Indeed, responders’ experience on this topic was heterogeneous, as depicted in Fig. 5. The possibility of down-titrating antihypertensive drugs in the post-operatory or during medical treatment, as well as the approach to dyslipidaemia and CV risk after surgery also widely differed among centres. Lastly, the glycaemic target in diabetic CS patients was controversial (see above). We used a linear regression analysis to assess whether the definitions or the assessing tools used by RCs were related to the reported outcomes after surgery. Only a trend to an inverse correlation between the number of tools used to assess hypertension (i.e., office measurements, 24-h ambulatory blood pressure monitoring and/or echocardiography) and hypertension recovery was found; the correlation presented low strength (*Supplementary Table 2*).

## Discussion

This survey evaluates the clinical experience on CS from 19 RCs across Europe accounting for an estimated pool of new diagnoses above 200 yearly and almost 1000 chronically cared patients yearly (Table 1). The low number of patients followed at some centers may depend on the rarity of the condition, on the high mortality of ectopic ACTH secreting tumours and adrenal cancer, on the discharge of benign unilateral adrenal forms and on the role of non-referral centers (*Supplementary materials*).

 Most of the experts agreed on a life-long monitoring for CS patients, although some of them considered discharge in specific settings, (e.g., benign unilateral adrenal CS cured after surgery). Although the discontinuation of monitoring appears reasonable in cases at very low risk, long-term consequences of cortisol exposure should be accounted and addressed irrespectively of the endocrinologist’s involvement in follow-up (e.g., general practitioner, other specialists). Indeed, all participants agreed that there is an increased CV risk in CS and the majority also acknowledged its persistence during time, irrespectively of remission status. Indeed, prior reports described persisting CV risk and complications for years following remission [6, 7] suggesting the need for long-term monitoring and management of modifiable CV risk determinants also in cured CS. The most recent consensus on CD does not address this issue specifically but recommends managing patients according to current guidelines for patients at high CV risk [5]. Based on this statement, an ideal approach would be the one reported in Tables 3, 4 [11–17]. Of note, the lack of condition-specific studies should be considered when assessing accountability of the physician for clinical decisions in this setting.

**Table 3:** Definition of a patient with high/very high cardiovascular (CV) risk in general population and in Cushing’s syndrome (CS)

Definitions in general population [11]	
Very high CV risk	High CV risk
$\surd$ Documented CVD $\surd$ DM with organ damage (i.e., microvascular complication), 3 major risk factors^a^or early onset– very prolonged duration (> 20 yrs) $\surd$ Severe CKD (eGFR < 30 mL/min/1.73m^2^) $\surd$ Calculated SCORE ≥ 10% for 10-year risk of fatal CVD $\surd$ FH with a major risk factor	$\surd$ Markedly elevated single risk factor^b^ $\surd$ DM without organ damage, with 1 major risk factor^a^ or prolonged duration (> 10 yrs) ⎫ Moderate CKD (eGFR 30–59 mL/min/1.73m^2^) $\surd$ Calculated SCORE 5–10% for 10-year risk of fatal CVD $\surd$ FH without a major risk factor
^a^Obesity, dyslipidaemia, age, hypertension, smoke	^b^BP > 180/110 mmHg, LDLc > 4.9 mmol/L (190 mg/dL)

Current suggested adaptation in CS [5]	
$\surd$ Active or remittent CS with multiple comorbidities or prior events falling in general population criteria for very high CV risk	$\surd$ Active CS $\surd$ CS in remission with persisting comorbidities falling in general population criteria for high CV risk

DM: diabetes mellitus; CKD: chronic kidney disease; eGFR: estimated glomerular filtration rate; SCORE: systematic coronary risk estimation; FH: familiar hypercholesterolemia; BP: blood pressure; LDLc: calculated low density lipoproteins

**Table 4:** Comorbidities management according to current guidelines (specific concomitant conditions could modify the management) compared to survey results considering Cushing’s syndrome (CS) at high cardiovascular (CV) risk

Management of a patient at high or very high CV risk according to general population guidelines		Current survey
***Diabetes mellitus***	2022 Joint Consensus report by ADA and EASD[12] 2023 ESC guideline[13]	*CS*
$\surd$ The treatment goal is usually set at 53 mmol/mol (7%) for glycosylated haemoglobin if safely reachable $\surd$ In patients at high CV risk or already established atherosclerotic complications (i.e., very high), treatment with GLP1 analogues and/or SGLT2 inhibitors should be initiated [14]		19/19 (100%) 3/19 (16%)
***Arterial hypertension***	2023 ESH guideline[15] 2024 ESC Guideline[16]	*CS*
$\surd$ In case of arterial hypertension (BP ≥ 140/90 mmHg) of any grade, irrespectively of CV risk, antihypertensive drugs should be started; for high-normal/elevated BP (130–139/85–89 mmHg) consider drug treatment on top of lifestyle interventions only in patients with very high CV risk (or in case of high CV risk with additional risk modifiers). The treatment goal should be a BP of 120–130/70–80 mmHg $\surd$ Irrespectively of CV risk, start with a two-drug combination of ACE inhibitors, ARBs, CCBs and thiazides/thiazide-like drugs due to their CV protection; use BBs in specific settings		Targets: Systolic BP 11/18 (61%) Diastolic BP 15/18 (83%) 15/19 (79%)
***Dyslipidaemia***	*2019 ESC/EAS guideline *[11]	*CS*
$\surd$ Patients at high and very high CV risk should be treated aiming to a 50% reduction in LDLc and to an absolute value < 1.8 mmol/L (70 mg/dL) and < 1.4 mmol/L (55 mg/dL) respectively; statins are the first-line treatment $\surd$ Aim to triglyceride’s level < 1.7 mmol/L (150 mg/dL)		8/19 (42%) *n.a.*
***Obesity***	2015 ES guideline [33]	*CS*
$\surd$ A pharmacological approach on top of lifestyle changes should be used in overweight patients (BMI ≥ 27 kg/m^2^) with comorbidities and in obese patients (BMI ≥ 30 kg/m^2^); a bariatric surgery should be considered in grade II obesity with comorbidities or if BMI ≥ 40 kg/m^2^		1/19 (5%)

ADA: American Diabetes Association; EASD: European Association for the Study of Diabetes; ESC: European Society of Cardiology; GLP: glucagon-like peptide; SGLT: sodium-glucose transporter; BP: blood pressure; ACE: angiotensin converting enzyme; ARBs: angiotensin receptor blockers; CCBs: calcium-channels blockers; BBs: beta blockers; EAS: European Atherosclerosis Society; LDLc: calculated low density lipoproteins; n.a.: not available; ES: Endocrine Society; BMI: body mass index

Notably, based on CV comorbidities clustering and established CV disease, many patients could be considered at high/very high CV risk independently of hypercortisolism, both during active disease and after cure (as the latter is not always associated with comorbidities resolution, see below). Based on the answers of the survey, also cases lacking “usual” definitions for high CV risk should receive careful management of CV risk factors (Tables 3 and 4). Participants reported careful risk stratification, that on top of CS-related comorbidities accounted for other factors such as smoking habit and familiar history that, if present, encouraged further investigations or cardiologist referral in line with the most recent consensus [5].

Most responders reported a relationship between CV risk and the degree of cortisol excess explaining the choice of most RCs to apply cortisol-lowering treatments in the preoperative period only in patients with severe hypercortisolism. Indeed, various reports addressed a significant perioperative mortality due to CV causes in CS patients [18, 19]making this approach reasonable despite the apparent lack of influence on the surgical outcome [20].

On top of clinical measurements, blood pressure was also assessed with the 24-h blood pressure monitoring, that could reveal masked hypertension due to the characteristic non-dipping phenotype of CS patients [21] and with echocardiography. In our survey the preferred antihypertensive treatment were angiotensin converting enzyme inhibitors, coherent with their known CV protective effect. Unsurprisingly, calcium channel blockers and angiotensin receptor blockers were other cited treatments as indicated by the most recent guidelines [15, 16]. Despite scarce data on CV protection for mineralocorticoid receptor antagonists (MRAs), 11% of responders considered them as the first-line option for hypertensive CS patients, probably to target the pseudo-hyperaldosteronism secondary to cortisol excess [22]. In line with this consideration, the European society of hypertension (ESH) suggested that MRAs could be considered as a first line treatment in cases presenting hypokalaemia. Vice versa, as thiazides/thiazide-like drugs can lead to hypokalaemia, their use should be restricted to patients presenting nephrolithiasis [23]. Treatment goals were in line with the current guidelines for most participants (< 130/80 mmHg), with a part of responders still correctly following the 2018 guidelines that suggested a stricter control (SBP < 120 mmHg) in young patients [24].

All responders recommended to assess glucose homeostasis at diagnosis and during follow-up. The risk of underdiagnosis previously advocated in literature [25] seems confirmed since only 26% of RCs performed an OGTT, while most relied on fasting glycaemia and HbA1c. Given the lack of patient-based study for CS related DM, current general recommendations have been borrowed from type 2 DM patients with metformin and GLP1 analogues as first line choices, thanks respectively to insulin sensitizing properties and to CV protection and anorectic effects. Conversely to what is recommended for DM patients at high CV risk, SGLT2 inhibitors should be used with caution since they can promote genitourinary tract infections [26]. In our survey, all participants preferred metformin as first-line treatment thanks to its benefit-cost ratio. In unoperated cases or persistent/relapsing disease physicians considered also other compounds with potentially more protective CV profile. Our survey did not delve into combination treatments and various centres reported local limitations in the prescription of novel drug categories with proven CV benefit. When antidiabetic therapy is started, a tendency towards strict control emerged, with many RCs aiming to a HbA1c fairly below the recommended 53 mmol/mol (7%). This cut off is related to prior literature addressing a U-shaped relationship between CV risk and HbA1c: an intensive HbA1c control was effective in reducing CV complications in some studies [27, 28] but this benefit was lost in case of lower target values leading to recurrent hypoglycaemia [29–31]. Nevertheless, with newer compounds allowing significant HbA1c decreases without the above-mentioned risk, more ambitious targets may be justified.

Dyslipidaemia in CS is characterized by increase of LDLc and triglycerides and reduction in high density lipoproteins. Most RCs suggested to regularly evaluate lipid profile in CS patients, both at diagnosis and during follow-up. Previous data showed that disease remission favoured lipid profile amelioration (Table 5); one study reported long-term impairment that may be due to residual overweight/obesity [6]. Most of RCs suggest lipid lowering treatments withdrawal after CS remission to reassess lipid profile and CV risk, but its timing varied widely (e.g., routinely 3 months after surgery, after various years of remission or in case of other comorbidities resolution). Especially the indications to delayed re-evaluation may be related to the awareness of prolonged increase in CV risk for CS patients, to the safety of lipid lowering agents available and to the increasing evidence favouring a “the lower the better” approach to dyslipidaemia [32]. Most RCs suggested to treat a LDLc above 3.9 mmol/L (150 mg/dL) with a target of 2.6 mmol/L (100 mg/dL) and to favour statin treatment. Therefore, assuming a high CV risk for CS patients, we registered a tendency to undertreat this comorbidity in current clinical practice, advocating further studies focusing on this topic.

As far as obesity was concerned, most RCs only evaluated the BMI, without examining body composition or waist to hip ratio (WHR) routinely. The latter is an easy to obtain parameter, that may prove useful in clinical practice: cured CD patients presented higher WHR than BMI-matched controls, suggesting a pivotal role of visceral obesity in their residual CV risk [6]. Persistent abnormal fat distribution (i.e., increased visceral fat) is indeed a recognized highly sensitive marker of increased CV risk. Irrespectively of the poor outcome for this comorbidity after remission, most participants preferred lifestyle interventions alone over the addition of anorectic drugs. This approach probably follows the limited availability of data on CV outcomes of anorectic drugs, although there are promising data on GLP1 analogues [33, 34] and more recently on the novel dual incretin agonist tirzepatide [35]. Regarding diabetic obese patients, a GLP1 analogue should be used according to current guidelines [13].

Sleeping apnoea is a possible complication of CS [36]but it is rarely inquired in clinical practice. Since performing polysomnography on a routine basis might be difficult, a possible pre-selection approach through dedicated questionnaires or ambulatory oximetry may aid in clinical practice.

Most RCs performed routine screening for CV and cerebrovascular disease and half of the participants repeated the screening regularly during follow-up. The preferred assessments were electrocardiography, echocardiography and carotid ultrasound. Whether this screening is cost effective may be debatable in asymptomatic patients. Nevertheless, electrocardiography is a basic screening procedure for DM- and hypertension-related organ damage [13, 15]and carotid ultrasound is justified in asymptomatic patients with multiple risk factors according to the 2023 ESVS guidelines [37]. The use of echocardiography is recommended as a part of an extended screening in hypertensive patients [15]: with CS patients presenting a higher degree of left ventricular hypertrophy than controls following pseudo-hyperaldosteronism related myocardial fibrosis [38] cardiac ultrasound has probably an additional value in risk stratification in this setting. Antiplatelet treatment was suggested only in case of documented vascular disease by most responders, in line with current guidelines [37, 39, 40]. Of note, CS induced cardiac alteration proved reversible upon its resolution [38, 41].

Participants experience on expected comorbidity reversal upon CS remission widely varied (Fig. 4), in line with actual data from literature (Table 5) [3, 6, 41–49]. Interestingly, Schernthaner-Reiter et al. found a negative correlation between the severity of hypercortisolism and persisting comorbidities that might depend on a delay in the diagnosis of mild cases and consequently longer exposure to cortisol excess causing irreversible modifications contributing to CV risk [46]. The heterogeneity in clinician’s responses to comorbidity reversal rates after remission could also have followed differences in defining and assessing the comorbidities. Regression analysis for glycemic and blood pressure’s outcomes after surgery only identified a trend to an inverse correlation between the number of tools used to assess hypertension and hypertension recovery. This finding could be expected, as a meticulous screening for residual hypertension may unveil additional cases reducing the proportion of complete post-surgical recovery. Moreover, independently of hypercortisolism, several other risk factors such as smoking, age, dietary habits, familial history may contribute to maintaining CV morbidity [50].

**Table 5:** Previous reports regarding comorbidities reversal after remission of Cushing’s syndrome (CS)

Study, year	Population, treatments used	Duration of remission	Total study patients	Arterial hypertension reversal	Glycaemic impairment reversal^a^	Overweight/obesity reversal^a^	Dyslipidaemia reversal
Fallo et al. [42]	CD and ACS, Surgery	12–60 months	54	72% (39/54)	n.a.	n.a.	n.a.
Colao et al. [6]^b^	CD, any	60 months	15	40% (4/10)	31% (4/13)	27% (11/15)	n.a.
Faggiano et al. [43]	CD, any^c^	12 months	25	44% (8/18)	40% (2/5)	38% (3/8)	HC 23% (3/13) HT 40% (2/5)
Gómez et al. [44]	CD and ACS, any	Months	71	75% (53/71)	n.a.	n.a.	n.a.
Pereira et al. [41]^d^	CD and ACS, any	12–18 months	15	33% (2/6)	40% (2/5)	n.a.	n.a.
Giordano et al. [45]^b^	CD and ACS, Surgery	12 months	29	38% (8/21)	67% (10/15)	27% (6/22)	35% (6/17)
Schernthaner-Reiter et al. [46]	CD and ACS, surgery	95 months (median)	118	36%	56%	44%	23%
Jha et al. [47]	CD and ACS, surgery	12 months	42	64% (27/42)	n.a.	n.a.	n.a.
Mondin et al. [3]^e^	CD, any	130.5 months (median)	126	60% (30/50)	40% (10/25)	32% (12/28)	39% (9/23)
Chihaoui et al. [48]	CD and ACS, surgery	12 months	75	58%	76%	n.a.	17%
Zhou et al. [49]^b^	ACS, surgery	38.5 months (median)	8	50%	0%	0%	0%

Note that in some articles: ^a^ overweight and prediabetes state were not included; ^b^ outcomes were calculated by difference in percentages assuming no new-onset comorbidity after surgery; ^c^ patients controlled by medical treatment were included; ^d^ outcomes were based on drug discontinuation; ^e^ data were derived by the unpublished original database. CD: Cushing’s Disease; ACS: adrenal CS; n.a.: not available; HC: hypercholesterolemia; HT: hypertriglyceridemia

Conversely, comorbidity reversal with cortisol lowering treatments was deemed unlikely by most, although an amelioration of comorbidity control is expected based on literature [51]. The ESH [23] stated that an improvement in blood pressure is observed upon hypercortisolism control in CS, independently of the specific drug used, although steroidogenesis inhibitors might cause hypertension worsening via pseudo-hyperaldosteronism caused by mineralocorticoid precursors accumulation. Regarding DM, a joint statement from Italian societies of Endocrinology and of Diabetes previously reported that an improvement of glycaemic profile is expected for all compounds except for pasireotide [52]; a recent phase IV study showed the important role of incretin-based antidiabetic drugs in this latter setting [53]. Ketoconazole and pasireotide proved effective in ameliorating lipid profile in CS, whilst mitotane was related to its worsening [54, 55]. Some CS treatment proved effective in weight management [55, 56].

Current guidelines for high CV risk patients paired with setting specific considerations can provide guidance on the ideal management of CS patients. Our survey highlights the awareness of physicians managing CS on issues related to metabolic and CV disease, but some gaps emerged. A larger use of provocative tests for studying hyperglycaemia is probably required. Physicians should also attempt to optimize treatments of dyslipidaemia and weight excess irrespective of disease phase. Since several CV risk factors seem to persist during disease remission, they should be adequately and promptly addressed. However, population-specific studies are required to identify the optimal management of CV and metabolic comorbidities of CS patients. Moreover, it would be advisable to improve awareness of these issues in non-endocrinologist colleagues following these patients, to provide an integrated care.

## Supplementary Information

Below is the link to the electronic supplementary material.

Supplementary Material 1

## Data Availability

All data generated or analysed during this study are included in this published article or in its *Supplementary Data*.
