# Supplementary material for: European survey on metabolic and cardiovascular risk in Cushing syndrome
Source: J Endocrinol Invest. 2025 Sep 6;48(11):2657–70. doi: 10.1007/s40618-025-02663-9 (PMC12602549; doi:10.1007/s40618-025-02663-9)
Supplement: Supplementary file 1 — Supplementary Material 1 [file 40618_2025_2663_MOESM1_ESM.docx]

**European survey on metabolic and cardiovascular risk in Cushing syndrome**

**Supplementary Data**

*Authors:* Alessandro Mondin^1,2^, Mattia Barbot^1,2^, Filippo Ceccato^1,2^, Alberto M Pereira^3,4^, Anna Angelousi^5^, Atanaska Elenkova^6^, Birute Zilaitiene^7^, Camilla Schalin-Jäntti^8^, Carlien De Herdt^9^, Christina Kanaka-Gantenbein^10^, Dominique Maiter^11^, Federico Gatto^12,13^, Gudmundur Johannsson^14^, Kirstine Stochholm^15^, Maria João Bugalho^16^, Mario Detomas^17^, Martin Reincke^18^, Meropi Toumba^19^, Roberta Giordano^20^, Stylianos Tsagarakis^21^, Susan M Webb^22^, Nienke R Biermasz^3^, Carla Scaroni^1,2^

*^1^ Department of Medicine-DIMED, University of Padova, Padova, Italy.*

*^2^ Endocrinology Unit, University Hospital of Padova, Padova, Italy*

*^.3^ Center for Endocrine Tumors Leiden, Department of Medicine, Division of Endocrinology, Leiden University Medical Center, Leiden, the Netherlands.*

*^4^ Pituitary Centre Amsterdam, Amsterdam, the Netherlands.*

*^5^ Unit of Endocrinology, First Department of Internal Medicine, Laikon Hospital, Center of Excellence of Endocrine Tumours, National and Kapodistrian University of Athens, 11527 Athens, Greece.*

*^6^ Department of Endocrinology, Faculty of Medicine, Medical University-Sofia, USHATE "Acad. Iv. Penchev", 2, Zdrave Str., 1431 Sofia, Bulgaria.*

*^7^ Institute of Endocrinology, Medical Academy, Lithuanian University of Health Sciences, 50161 Kaunas, Lithuania.*

*^8^ Endocrinology, Abdominal Center, Helsinki University Hospital and University of Helsinki, ENDO-ERN (European Reference Network on Rare Endocrine Conditions), Helsinki, Finland*

*^9^ Department of Endocrinology, Diabetology and Metabolic Diseases, Antwerp University Hospital, Antwerp, Belgium.*

*^10^ Division of Endocrinology, Diabetes and Metabolism and Aghia Sophia ENDO-ERN Center for Rare Pediatric Endocrine Disorders, First Department of Pediatrics, Medical School, Aghia Sophia Children's Hospital, National and Kapodistrian University of Athens, 11527 Athens, Greece.*

*^11^ Department of Endocrinology and Nutrition, Cliniques Universitaires Saint-Luc UCL, Bruxelles, Belgium.*

*^12^ Endocrinology Unit, Department of Internal Medicine and Medical Specialties, University of Genova, 16132 Genova, Italy.*

*^13^ IRCCS Ospedale Policlinico San Martino, 16132 Genova, Italy.*

*^14^ Department of Internal Medicine and Clinical Nutrition, Institute of Medicine, Sahlgrenska Academy, University of Gothenburg & Department of Endocrinology, Sahlgrenska University Hospital, Gothenburg, Sweden.*

*^15^ Department of Internal Medicine and Endocrinology, Aarhus University Hospital, Aarhus, Denmark.*

*^16^ Serviço de Endocrinologia, ULS Santa Maria, Lisboa, Portugal*

*^17^ Department of Internal Medicine I, Division of Endocrinology and Diabetes, University Hospital, University of Würzburg, 97080 Würzburg, Germany.*

*^18^ Medizinische Klinik und Poliklinik IV, LMU University Hospital, LMU Munich , Munich, Germany.*

*^19^ American Medical Center, Strovolos 2047 & School of Medicine, University of Nicosia, 2414 Nicosia, Cyprus.*

*^20^ Department of Biological and Clinical Sciences, University of Turin, Turin, Italy.*

*^21^ Department of Endocrinology, Diabetes and Metabolism, European Reference Network on Rare Endocrine Conditions (ENDO-ERN), Evangelismos Hospital, 10676, Athens, Greece.*

*^22^ Department of Medicine, Univ Autonoma Barcelona; Research Center for Pituitary Diseases, Institut de Recerca Sant Pau (IIB-Sant Pau) and CIBERER Unit 747, ISCIII; Department of Endocrinology, Hospital S Pau, Barcelona, Spain.*

***Supplementary Table 1.*** *Survey questions. Domains included are definitions and epidemiology (red), cardiovascular risk (green), arterial hypertension (blue), glucose metabolism (yellow), lipid metabolism (purple), vascular disease (orange), obesity (grey) and miscellany (green water). MC: multiple choices; OF: open field; CS: Cushing’s syndrome; CD: Cushing’s Disease; EAS: ectopic ACTH secretion; b: benign; m: malignant; ACS: adrenal Cushing’s syndrome; OGTT: oral glucose tolerance test; usPCR: ultra-sensitive C-reactive protein; BNP: b-type natriuretic peptide; GFR: glomerular filtration rate; Na: sodium; K: potassium; Mg: Magnesium; Ca: calcium; HOMA-IR: homeostatic model assessment for insulin resistance; HbA1c: glycosylated haemoglobin; GLP: glucagon like peptide; DDP: dipeptidyl peptidase; SGLT: sodium glucose transporter; LDL: low density lipoproteins; PCSK: proprotein convertase subtilisin/kexin; CT: computed tomography.*

| ***1. How do you define a new patient? (MC, OF)*** |
| --- |
| Treatment naïve patients |
| Patients not previously seen by the reference center |
| Any patient with an exceeding interval between the last and the present consultation depending on the health record of the reference center (for instance more than 12 months) |
| Patients with recurrent disease after initial remission |
| Others, please specify |
| ***2. How do you define a patient under chronic care? (OF)*** |
| Patients under active treatment at the reference center |
| Patients with previous treatment at the reference center (e.g. patients in complete remission after treatment) |
| Patients with previous treatment currently under affiliated centers referred to the reference center for a single consultation only, diagnostic tests, or for specific procedure |
| Others, please specify |
| ***3.1 Do you have a specific database containing clinical characteristics of patients with CS?*** |
| Yes/No |
| ***3.2 If yes, what kind of database? (OF)*** |
| ***3.3 Do you use the ERCUSYN registry?*** |
| Yes/No |
| ***4.  What is the number of patients newly diagnosed with the following CS subtypes in your center in 2020 and 2021? (OF)*** |
| Number of CD/EAS/b-ACS/m-ACS for 2020 and 2021 |
| ***5.  What is the number of patients with the following CS subtypes under chronic care in your center in 2020 and 2021? (OF)*** |
| Number of CD/EAS/b-ACS/m-ACS for 2020 and 2021 |
| ***6.1 Does discharge of follow-up depend on the aetiology of CS?*** |
| Yes/No |
| ***If yes, please specify (OF)*** |
| ***6.2 Is discharge of follow-up regarding patients with CS related to remission status? (MC)*** |
| Discharge upon remission |
| Discharge X months/years in remission |
| Not related to remission status, life-long follow-up |
| Not related to remission status; patients are discharged from follow up when Y |
| ***If X and/or Y, please specify (OF)*** |
| ***7. Do you consider CS a condition at high risk for cardio-metabolic diseases?*** |
| Yes/No |
| ***8.  When, according to you, patients with CS have increased CV risk factors? (MC, OF)*** |
| During active phase before diagnosis |
| While waiting for surgery |
| During medical therapy with cortisol lowering medication |
| Until 1 year after effective surgery |
| Until 5 years after effective surgery |
| They will be always at risk |
| Others, please specify |
| ***9. In your opinion, cardiovascular risk is related to the severity of hypercortisolism?*** |
| Yes/No |
| ***10. What parameter do you use to stratify the degree of hypercortisolism? (MC, OF)*** |
| 24h-Urinary free cortisol |
| Late night serum cortisol |
| Morning/late night salivary cortisol ratio |
| Others, please specify |
| ***11. Which of the following biochemical exams are included in baseline analysis of patients with CS? (MC, OF)*** |
| Fasting glycaemia, 2h post-prandial glycaemia, HbA1c, fasting insulin, OGTT, Lipid profile, Microalbuminuria, usPCR, Uric acid, BNP, complete blood count, Renal function (GFR), Ions (Na, K, Mg, Ca), and/or Others, please specify |
| ***12. Arterial hypertension - How do you assess it? (MC)*** |
| Office blood measurement (one measurement) |
| Office blood measurement (mean of 3 measurements) |
| Sitting and supine blood pressure assessment |
| Arterial blood pressure monitoring (24h) |
| Patients self-monitoring |
| Echocardiography |
| Not routinely assessed |
| ***13. Arterial hypertension - When do you assess it? (MC, OF)*** |
| At CS diagnosis |
| 3-6 months after surgery (pituitary or adrenal or NET) |
| Regularly during medical treatment only for persistent/recurrent CS or not amenable for surgery (specify the time of regular follow-up) |
| At diagnosis and regularly during follow-up in both remittent and persistent CS |
| Others, please specify |
| ***14. Arterial hypertension - Which is the target to achieve? (OF)*** |
| Systolic < 120 diastolic < 80 mmHg? |
| Systolic < 130 diastolic < 80 mmHg? |
| Systolic < 140 diastolic < 90 mmHg? |
| Others, please specify |
| ***15. Which is your first-choice treatment for hypertension in CS? (OF)*** |
| Angiotensin converting enzyme inhibitors, Angiotensin receptor blockers, Mineralocorticoid receptor antagonists, Calcium-channel blockers, Beta-blockers or Others, please specify |
| ***16. How many patients reduce drugs for hypertension after effective surgery? (OF)*** |
| <25%, 25-50%, 50-75%, > 75% or Others, please specify |
| ***17. In your experience, how many patients experience complete recovery from hypertension after surgery? (OF)*** |
| 100%, 75%, 50%, 25%, or Others, please specify |
| ***18.1 How many patients reduce drugs for hypertension while on cortisol lowering medications? (OF)*** |
| <25%, 25-50%, 50-75%, > 75% or Others, please specify |
| ***18.2 Does the presence of arterial hypertension modify your choice of cortisol-reducing medication for patients with recurrent/persistent disease?*** |
| Yes/No |
| ***If yes, please specify (MC)*** |
| Ketoconazole, Metyrapone, Cabergoline, Pasireotide, Mitotane, Osilodrostat, Levo-ketoconazole,  Mifepristone, Etomidate and/or Combined therapy |
| ***If Combined therapy, please specify (OF)*** |
| ***19. Do you routinely evaluate glucose metabolism in CS patients?*** |
| Yes/no |
| ***20. Do you always ask for familiar history of diabetes mellitus to your CS patients?*** |
| Yes/no |
| ***21. Glucose metabolism - How do you assess it? (MC)*** |
| Morning fasting serum glucose |
| 2-hours post-prandial glucose |
| HOMA-IR |
| 2-hours oral glucose tolerance test for glucose and insulin |
| HbA1c |
| Not assessed |
| ***22. Glucose metabolism - When do you assess it? (MC, OF)*** |
| At CS diagnosis |
| 3-6 months after surgery (pituitary or adrenal or NET) |
| Regularly during medical treatment only for persistent/recurrent CS or not amenable for surgery (specify the time of regular follow-up) |
| At diagnosis and regularly during follow-up in both remittent and persistent CS |
| Others, please specify |
| ***23. Which is your first-choice treatment for CS patients with diabetes at diagnosis? (OF)*** |
| Metformin, GLP-1 analogues, DDP-IV inhibitors, SGLT2 inhibitors, insulin, or Others, please specify |
| ***24. Which is your first-choice treatment for diabetes in CS for during persistent/recurrent CD or not-amenable for surgery? (OF)*** |
| Metformin, GLP-1 analogues, DDP-IV inhibitors, SGLT2 inhibitors, insulin, or Others, please specify |
| ***25.1 Are there any restrictions to the prescription of some antidiabetic classes in your Country?*** |
| Yes/no |
| ***25.2 If Yes to the above question, if you could prescribe any anti-diabetic medication without them, what would your first choice be? (OF)*** |
| Metformin, GLP-1 analogues, DDP-IV inhibitors, SGLT2 inhibitors, insulin, or Others, please specify |
| ***25.3 In your experience, how many patients experience complete recovery from diabetes after surgery? (OF)*** |
| 100%, 75%, 50%, 25%, or Others, please specify |
| ***25.4 Which is the target to achieve? (OF)*** |
| HbA1c below 42, 48, 53, 58 mmol/l or Others, please specify |
| ***25.5 Does the presence of DM modify your choice of cortisol-reducing medication for patients with recurrent/persistent disease?*** |
| Yes/no |
| ***If yes, please specify (MC)*** |
| Ketoconazole, Metyrapone, Cabergoline, Pasireotide, Mitotane, Osilodrostat, Levo-ketoconazole,  Mifepristone, Etomidate and/or Combined therapy |
| ***If Combined therapy, please specify (OF)*** |
| ***26. Do you routinely assess lipid metabolism in CS?*** |
| Yes/no |
| ***27. Lipid metabolism - When do you assess it? (MC, OF)*** |
| At CS diagnosis |
| 3-6 months after surgery (pituitary or adrenal or NET) |
| Regularly during medical treatment only for persistent/recurrent CS or not amenable for surgery (specify the time of regular follow-up) |
| At diagnosis and regularly during follow-up in both remittent and persistent CS |
| Others, please specify |
| ***28. In which cases do you prescribe lipid lowering medications in CS? (OF)*** |
| Total cholesterol > 200 mg/dl |
| LDL cholesterol > 150 mg/dl |
| Triglycerides > 150 mg/dl |
| Always in CS |
| Others, please specify |
| ***29. What is your treatment goal for dyslipidaemia in CS? (OF)*** |
| Total cholesterol <200 mg/L, LDL cholesterol < 100, 70, 55 or 40 mg/L, Triglycerides < 150 mg/dl, or Others, please specify |
| ***30. Which is your first-choice treatment for dyslipidaemia in CS? (OF)*** |
| Dietary indications and physical exercise, statin, statin + ezetimibe, fenofibrate, PCSK-9 inhibitors, or Others, please specify |
| ***31. After surgical remission do you withdraw lipid lowering medication and reassess cardiovascular risk?*** |
| Yes, within 3 months after successful surgery and re-test the patient |
| Yes, but only after 1 year of CS remission |
| I consider suspending the treatment only if other comorbidities disappeared |
| No, I keep the medications |
| ***32. Do you consider CS a condition at high risk for cardio- and cerebro-vascular diseases?*** |
| Yes/no |
| ***33. Do you routinely screen your patient with CS for cardio- and cerebrovascular diseases?*** |
| Yes, only active CS |
| Yes, all CS |
| No |
| Yes, but only patients with known cardiovascular risk factors |
| Yes, but only patients with previous cardio- and cerebrovascular events |
| ***34. Which of the following exams do you recommend to your CS patients? (MC, OF)*** |
| Electrocardiogram |
| Carotid US |
| Echocardiography |
| Cardiac MR |
| Coronarography study (CT or angiographic) |
| Cerebral magnetic angiography |
| Others, please specify |
| ***35. Vascular disease - When do you assess it? (MC, OF)*** |
| At CS diagnosis |
| 3-6 months after surgery (pituitary or adrenal or NET) |
| Regularly during medical treatment only for persistent/recurrent CS or not amenable for surgery (specify the time of regular follow-up) |
| At diagnosis and regularly during follow-up in both remittent and persistent CS |
| Others, please specify |
| ***36. Do you consider antiplatelet therapy for CS?*** |
| Yes, in all cases |
| Only in diabetic CS |
| Only in CS with documented vascular disease |
| No, due to bleeding concerns |
| ***37. How do you evaluate obesity at your Center? (OF)*** |
| Body mass index |
| Waist to hip ratio |
| Body composition through bioelectrical impedance analysis |
| Body composition through total body DXA scan |
| Others, please specify |
| ***38. Obesity is frequently associated to CS; in your experience how many patients remain overweight or obese after effective surgery compared to baseline? (OF)*** |
| 100%, 75%, 50%, 25%, or Others, please specify |
| ***39. In patients controlled by medical therapy how many patients remain overweight or obese compared to pre-treatment condition? (OF)*** |
| 100%, 75%, 50%, 25%, or Others, please specify |
| ***40. How do you address obesity in patients in remission?*** |
| Diet indication and physical exercise or Drugs |
| ***41. Do you prescribe cortisol lowering medication prior to surgery to better control cortisol related complication such as hypertension, diabetes, obesity? (OF)*** |
| Yes, routinely |
| Yes, but only in patients with severe Cushing’s syndrome (high cortisol levels) |
| Yes, but only in patients with previous cardiovascular event |
| No, never |
| Others, please specify |
| ***42. Do you assess sleep apnoea in active CS patients?*** |
| Yes/no |
| ***43. If yes, how do you evaluate it?*** |
| Self-reported |
| Epworth questionnaire |
| Polysomnography |
| Others, please specify |
| ***44. Sleep apnoea - When do you assess it? (OF)*** |
| Only during phase of disease |
| Only after surgical remission |
| During medical therapy in CS |
| Periodically |
| ***45. In your experience pre-treatment with cortisol lowering medication can lower the risk of cardiovascular event in the perioperative period?*** |
| Yes/no |
| ***46. What is the cortisol lowering medication you usually prescribe preoperatively to prepare your patients to surgery?*** |
| Ketoconazole, Metyrapone, Cabergoline, Pasireotide, Mitotane, Osilodrostat, Levo-ketoconazole,  Mifepristone, Etomidate and/or Combined therapy |
| ***If Combined therapy, please specify (OF)*** |
| ***47. Do you always ask for smoking habits to your CS patients?*** |
| Yes/no |
| ***48. The presence of these risk factors (smoking and familiar history of cardiovascular disease) independent from the disease itself, change your approach to the patients?*** |
| Yes, I would perform more instrumental exams (echocardiogram, etc.) |
| Yes, I would pre-treat the patients with cortisol lowering medication |
| Yes, I would recommend cardiac evaluation |
| No, I would send the patient immediately to surgery |
| ***49. Which of the following biochemical exams are included in your follow-up? (MC, OF)*** |
| Fasting glycaemia, HbA1c, fasting insulin, OGTT, Lipid profile, Microalbuminuria, usPCR, BNP, Complete blood count (neutrophilia and thrombocythemia), Others, please specify |

Expert centers, in addition to routine clinical practice, have their local or national policy of management, that may affect the definition of “new” and “chronically cared” patients. The definition of a new CS patient mainly referred to cases that have not been evaluated by the reference centre yet (16/19, 84%) or to treatment naïve patients (13/19, 68%). Other less common definitions were patients experiencing recurrence (n=3) and patients not recently evaluated at the reference centre (n=2). Regarding the definition of a CS patient under chronic care, most of responders (14/19, 74%) addressed those receiving active treatment or undergoing regular follow-up at the reference centre. Some RCs considered as chronically cared all subjects previously treated at the centre independently of where follow up is currently performed; on the contrary some responder included patients routinely followed by affiliated centres after treatment only if referred to the reference centre for consultation, diagnostic tests and/or procedures (11% each). One centre responded including both actively and previously treated patients on ongoing follow-up.

***Supplementary Figure 1.*** *Database compilation across centers participating in the survey. CS: Cushing’s Syndrome; ERCUSYN: European register on Cushing’s syndrome.*


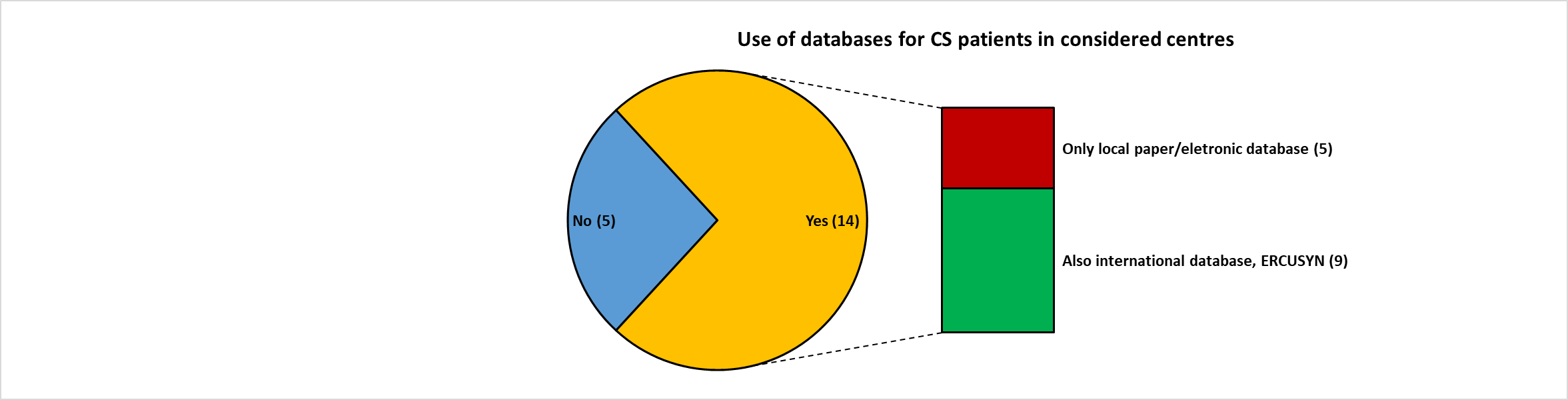


***Supplementary Figure 2.*** *Biochemical work-up for CS at diagnosis (blue) and during follow-up (green). CS: Cushing’s syndrome; na: not available; eGFR: estimated glomerular filtration rate; Na: sodium; K: potassium; Ca: calcium; OGTT: oral glucose tolerance test; BNP: b-type natriuretic peptide; us-PCR: ultra-sensitive reactive protein C.*

*
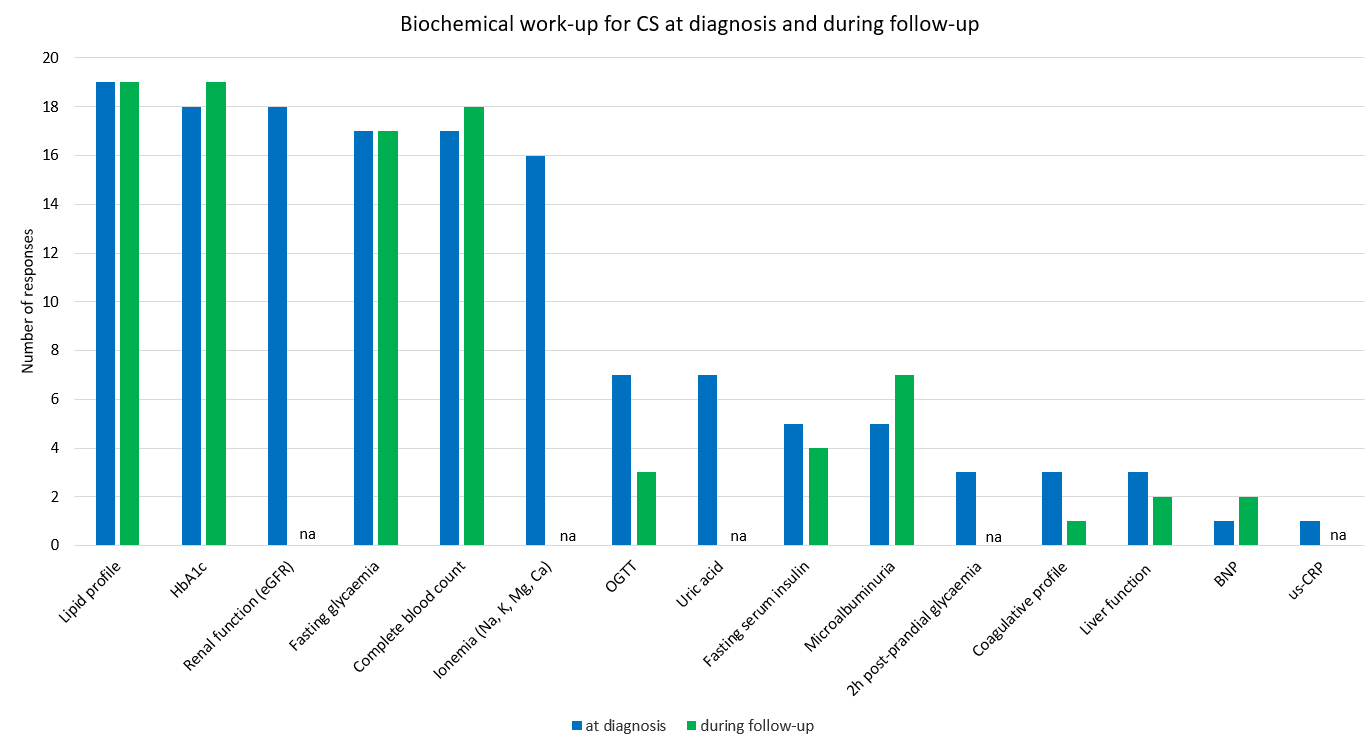
*

***Supplementary Figure 3.*** *Tools to stratify hypercortisolism degree according to survey responders. UFC: urinary free cortisol; LNSC: late-night salivary cortisol; MSC: midnight salivary cortisol; EMSC: early-morning salivary cortisol; DST: dexamethasone suppression test. * respectively clinical picture (n=1) and serum cortisol/ACTH levels (n=1).*

***Supplementary Figure 4.*** *Preferred means (A) and timing (B) to screen vascular disease according to participants. MRI: magnetic resonance imaging; CT: computed tomography; US: ultrasound; ECG: electrocardiogram; CS: Cushing’s syndrome; NET: neuro-endocrine tumor. * choice based on age and comorbidities (n=1) or on physical examination and prior medical history (n=1).*

***Supplementary Figure 5.*** *Proportion of centers choosing cortisol lowering treatments based on arterial hypertension (53%) in red, or on impaired glucose homeostasis (63%) in blue. For each category the preferred drugs (multiple choices) are reported. * 3 participants suggested a combination of ketoconazole and metyrapone for treating hypertensive patients; 2 centers advised using a combination of ketoconazole and metyrapone for treating diabetic CS patients, one of them also considered a ketoconazole plus cabergoline regimen.*

*
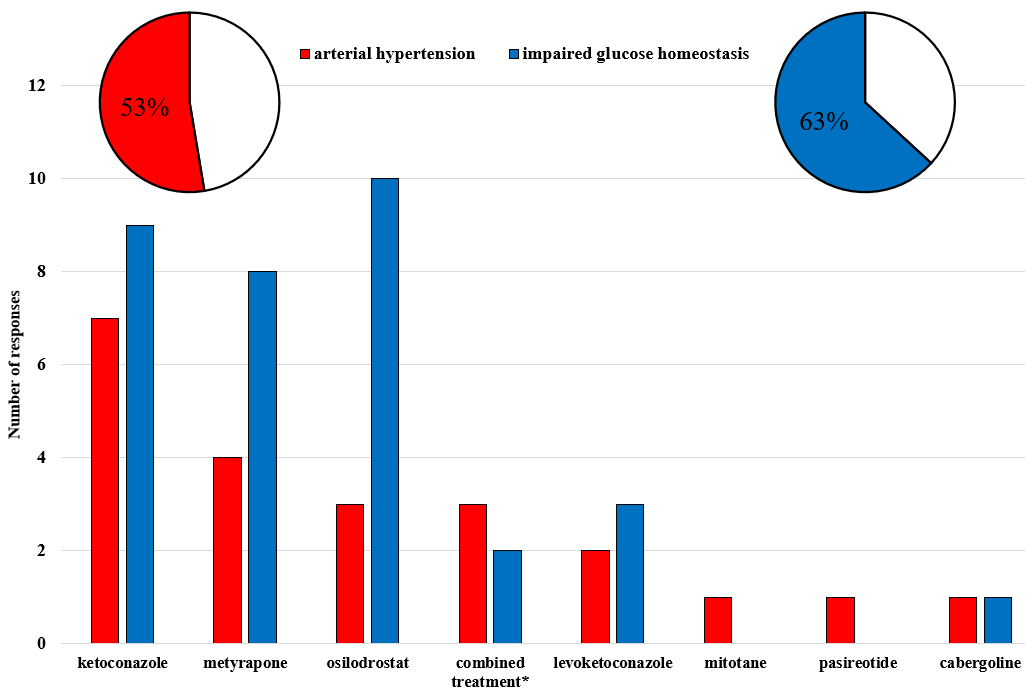
*

***Supplementary Table 2.*** *Simpler linear regression analysis for predictors of comorbidities outcome after surgery. R: Pearson coefficient; SBP: systolic blood pressure; n.a.: not available. * office measurements, 24-h ambulatory blood pressure monitoring and/or echocardiography. # SBP threshold for hypertension set at 120, 130 or 140 mmHg. £ fasting glycaemia, glycosylated haemoglobin and/or post prandial measurements.*

| Outcome after surgery | Predictor | R | R^2^ | p | Correlation | Strength |
| --- | --- | --- | --- | --- | --- | --- |
| Arterial hypertension recovery | Number of tools for assessment * | 0.384 | 0.148 | 0.058 | Trend to inverse correlation | low |
| Arterial hypertension recovery | Definition based on SBP # | 0.096 | 0.009 | 0.352 | none | n.a. |
| Antihypertensive drugs reduction | Number of tools for assessment * | 0.030 | 0.001 | 0.452 | none | n.a. |
| Antihypertensive drugs reduction | Definition based on SBP # | 0.100 | 0.010 | 0.348 | none | n.a. |
| Glucose homeostasis recovery | Number of tools for assessment £ | 0.014 | 0.000 | 0.479 | none | n.a. |
